# Health-related quality of life and radiological and functional lung changes of patients with COVID-19 Pneumonia 3 and 10 months after discharge

**DOI:** 10.1186/s12890-023-02520-6

**Published:** 2023-06-27

**Authors:** Cristina De Juana, Susana Herrera, Silvia Ponce, Sergio Calvache, Loubna Dahmazi, Raffaele Vitale, Alberto José Ferrer, Verónica Valentín, Marta Acosta, Irene López, Eva Martínez-Moragón, Respiratory medicine Unit of Universitary Hospital Doctor Peset of Valencia (Spain)

**Affiliations:** 1 Department of Respiratory Medicine, Universitary Hospital Doctor Peset of Valencia, Valencia, Spain; 2 University of Lille, CNRS, LASIRE, Lille, France; 3 Polytechnic University of Valencia, Valencia, Spain

**Keywords:** COVID-19, Health-related quality of life, Follow-up radiological findings and pulmonary function

## Abstract

**Background:**

Few studies have evaluated the long-term impact on health-related quality of life (HRQoL) in patients who have been hospitalized for COVID-19 pneumonia. Specific follow-up should be carried out to detect and treat possible pulmonary abnormalities, and the worsening of HRQoL should be estimated to target necessary resources for care of these patients after acute phase. The objective was to know the impact on HRQoL of patients who have been admitted for COVID-19 pneumonia, and to evaluate the clinical-radiological and functional changes of patients who have overcome COVID-19 pneumonia at 3 and 10 months of follow-up.

**Methods:**

Prospective observational study of patients who required hospitalization for COVID-19 pneumonia between April and December 2020. All patients filled out the EuroQol five-dimension (EQ-5D) questionnaire with the EuroQol Visual Analogue Scale (E-VAS) for self-assessment of health status. Respiratory function tests and chest X-ray were carried out at 3 and 10 months of follow-up.

**Results:**

61 patients were included in the study. The need for ventilatory support was associated with anxiety/depression on the EQ-5D scale, as well as patients admitted to the intensive care unit (ICU). The mean EQ-5D and E-VAS index scores decreased with hospitalization time, the number of days spent in intermediate respiratory care unit (IRCU) and the level of dyspnoea at the beginning of the hospitalization period. Pulmonary sequelae were observed in 25 patients (41%) at 3 months and 17 (27.9%) at 10 months. Patients improve their forced vital capacity (FVC) by 196 ml (p = 0.001) at 10 months as well as 9% in diffusing capacity of lung for carbon monoxide (DLCO) (p = 0.001) at 10 months. DLCO was found to be correlated to lymphopenia and time spent in IRCU. Low FVC values were detected 10 months after discharge for subjects exhibiting high levels of dyspnoea at 3 months after discharge.

**Conclusions:**

Hospitalization for COVID-19 pneumonia affects the HRQoL of patients, with greater anxiety/depression in those who were more serious affected and are younger. A significant percentage of patients present fibrotic abnormalities and lung function impairment at the first and second follow-up after discharge.

## Background

Severe acute respiratory syndrome coronavirus 2 (SARS-CoV-2) had led to coronavirus disease 2019 (COVID-19) and a current pandemic that represents a critical ongoing global healthcare problem [1].

The long-term complications of COVID-19 pneumonia are starting to emerge; in fact, data from previous coronavirus outbreaks such as severe acute respiratory syndrome (SARS) and Middle East respiratory syndrome (MERS) suggest that some patients will experience long-term complications of the infection [2].

After acute respiratory distress syndrome (ARDS) regardless of its origin, patients frequently show several functional impairments across biopsychosocial domains [3]. In recent years, HRQoL has payed worldwide attention, and several multidimensional health status classifications have been increasingly used to describe and evaluate the quality of life [4].

Regarding SARS-CoV-2, recent research has demonstrated that nearly half of discharged patients show residual abnormalities on radiological images [5]. Furthermore, this study showed that in early convalescence (1 month after discharge), approximately three-quarters of patients with COVID-19 demonstrated pulmonary function impairment, represented most frequently again by declines in DLCO [6]. Another publication on patients with noncritical disease demonstrated that a considerable proportion of COVID-19 survivors showed radiologic (70%) and pulmonary function (25%) abnormalities 3 months after discharge [7].

Therefore, these patients should be followed up to detect and manage pulmonary sequelae and functional impairment [7]. With the anticipation of potential long-term sequelae after COVID-19, follow-up strategies have been proposed by several groups worldwide [8, 9].

Here, we aimed to present the temporal trends in physical and respiratory outcomes over 10 months in a prospective cohort of patients hospitalized with severe COVID-19 pneumonia.

## Methods

In this prospective study were approached for study participation patients with COVID-19 pneumonia discharged between April and December 2020 from Doctor Peset University Hospital, a tertiary hospital in Valencia, Spain.

Clinical data for acute phase, which was defined as the period between hospital admission and discharge, were retrieved from electronic medical records, including demographic and clinical characteristics (age, sex, comorbidities, time of admission, need for intensive care, need of ventilatory support, need of oxygen therapy after discharge, symptoms report), chest images and laboratory test results. Additionally, COVID-19 Severity Index [10] was included. Also known as Sum Score, it is obtained by adding the digits corresponding to the levels of the 5 dimensions in each state of health.

All participants were assessed at 3 and 10 months after discharge in an outpatient clinic consultation by trained medical staff. During the visit at 3 months follow-up, HRQoL, dyspnoea, chest X-ray and standardized pulmonary function tests were evaluated; whereas at 10 months follow-up visit, dyspnoea, chest X-ray and pulmonary function tests were evaluated.

### Evaluation of quality of life

Participants were asked to complete the EQ-5D questionnaire and E-VAS index [10] at 3-months follow-up after discharge.

EQ-5D scale was used in order to value quality of health state defined by the EQ-5D index. It measures five dimensions of health: mobility, self-care, usual activities, pain/discomfort, and anxiety/depression, as well as overall health rated on a E-VAS [11, 12].

### Imaging analysis and score index in chest RX

All chest radiographs (X-ray) were analyzed by two radiologists specialized, blind to the clinical information, except knowing that all were confirmed cases by positive SARS-COV2-test.

The affectation is defined as:

- Interstitial involvement: reticular interstitial pattern and small ill-defined opacities.
- Faint opacities: areas of condensation in ground glass / discrete density, which do not erase vascular structures.
- Alveolar infiltrates: alveolar condensation with imprecise borders.
- The modified quantitative radiological scoring system for radiological lung involvement [13] includes two steps in image analysis:

1) The first step consists of dividing the lungs into 6 zones on the chest X-ray:

- Upper areas (A and D): above the lower border of the aortic arch.
- Middle areas (B and E): between the inferior border of the aortic arch and the inferior border of the right inferior pulmonary vein (that is to say, comprising the region of the pulmonary hila).
- Inferior zones (C and F): between the lower border of the right inferior pulmonary vein and the diaphragm (that is, the pulmonary bases).

2) The second step consists of assigning a score (from 0 to 3) to each of these areas, based on the existing pulmonary abnormalities on the chest X-ray, as follows:

- Score 0: no pulmonary abnormalities
- Score 1: interstitial involvement
- Score 2: faint opacities (equivalent to “ground glass” on CT)
- Score 3: alveolar infiltrates

Scores for the 6 lung zones are added together to obtain an overall "CXR SCORE" (ranging from 0 to 18).

This score system was carried out both for the evaluation of the findings of COVID-19 infection in the chest X-ray at acute phase and for the control chest X-ray in the first and second follow-up.

### Pulmonary function tests

Pulmonary function tests were done according to American Thoracic Society (ATS)–and European Respiratory Society (ERS) guidelines [14, 15]. The following parameters were measured: spirometry; diffusing capacity of the lungs for carbon monoxide (DLCO).

### Statistical analyses

The underlying relations linking the clinical data for acute phase to the EQ-5D life quality indices, dyspnoea and standardized pulmonary functions levels and chest X-ray outcomes were assessed by means of Partial Least Squares regression (PLS) [16]. PLS is a statistical predictive approach that aims at modelling such intrinsic relationships in a multivariate fashion by maximizing the covariance between set of regressors and one or multiple *response* variables. In this study, the following two scenarios were envisioned and explored. Firstly, the effect of the demographic characteristics, clinical parameters and laboratory test results (regressors) on the EQ-5D life quality indices, dyspnoea level, standardized pulmonary functions level and chest X-ray outcomes at 3 months after discharge (responses) was evaluated. Thereafter, the influence of the demographic characteristics, clinical parameters, laboratory test results as well as of the EQ-5D life quality indices, dyspnoea level, standardized pulmonary functions level and chest X-ray outcomes at 3 months after discharge (regressors) on the results of the outpatient clinic consultation carried out after 10 months from discharge (responses) was studied. It is important to notice that EQ-5D life quality indices were all recodified as binary variables (absence or presence of problems) and that no EQ-5D questionnaire was answered 10 months after discharge. Three additional aspects are worth to be mentioned here:

1. every individual response variable was accounted for separately and independently, *i*.*e*., a single PLS model per response variable was fitted.
2. PLS model parameters were optimized through a leave-one-patient-out cross-validatory procedure;
3. the statistical significance of the effect of the various regressors on the considered response was estimated by jackknifing. Variables for which such an effect was found not to be statistically significant (p-value > 0.05) were iteratively removed until reduced PLS models encompassing a maximum number of 20 predictors were obtained.

The predictive performance of the final models was determined in terms of Root Mean Square Error in Cross-Validation (RMSECV), when numerical responses were analyzed, or cross-validated Area Under the Receiver Operating Curve (AUROC), when categorical responses were analyzed. The validity of the results yielded by the outlined strategy was finally confirmed via distinct statistical univariate assessments, depending on the nature of both the regressor and the response at hand: linear regression for numerical predictors and responses, one-way ANOVA for categorical predictors and numerical responses, Student’s *t*-test and logistic regression for numerical predictors and categorical responses, χ^2^ test and logistic regression for categorical predictors and responses.

Furthermore, potential variations between clinical parameters measured 3 months after discharge and 10 months after discharge were assessed by means of paired-sample *t*-testing (for numerical descriptors) and χ^2^ test (for categorical descriptors).

## Results

A total of 61 patients were included in the study. Patients were followed up for a median of 101 days (92–138; median) over 3 months, and 291 (261–316) over 10 months. The demographic and clinical characteristics of participants are shown in Table 1. Most patients had never smoked (62,3%). Their most common comorbidities were hypertension (50,8%) and dyslipidemia (44,3%) followed by diabetes (21,3%). During hospitalization, 25 of them (40,3%) required ventilatory support (non-invasive ventilation, invasive ventilation and high flow oxygen therapy), 17 participants (27,4%) were admitted to the ICU and 27 (43,5%) to the IRCU. The median duration of hospital stay was 20,9 (10–98) days and time exclusively in the ICU was 14,2 (8–40) days. Radiological findings in the acute phase showed that the lung areas most affected after discharge were the middle lobe and the lingula, followed by the lower lobes.

**Table 1:** Characteristics of enrolled patients

	Sample size *n* = 61
Age	65 (36–85)
Sex	
Male	37 (60.7%)
Female	24 (39.3%)
Smoking habits	
Never smoked	38 (62.3%)
Ex- smoker	21 (34.4%)
Current smoker	2 (3.3%)
Comorbidities	
Diabetes mellitus	13 (21.3%)
Hypertension	31 (50.8%)
Dyslipidemia	27 (44.3%)
OSA (obstructive sleep apnoea)	8 (13.1%)
Asthma	5 (8.2%)
COPD	5 (8.2%)
Chronic respiratory failure	2 (3.3%)
Continuous Home Oxygen Therapy	4 (6.6%)
Hospitalization	
Supplemental oxygen (nasal cannula)	17 (27.9%)
Supplemental oxygen (Venturi mask)	7 (11.5%)
Ventilatory support	
(NIMV, MV, High flow oxygen therapy)	25 (41,0%)
ICU admission	17 (27,9%)
IRCU admission	27 (44,3%)
Length of hospital stay (days; median)	20.4 (0–108)
Length of ICU (days; median)	3.5 (0–48)
Length of IRCU (days; median)	6.9 (0–64)
Dyspnea (mMRC scale)	
0	35 (57.4%)
1	15 (24.6%)
2	8 (13.1%)
3	3 (4.9%)
4	0 (0.0%)
Rx acute phase	
Rx A (RUL)	
Without abnormalities	51 (83.6%)
Interstitial thickening or reticulation	2 (3.3%)
Small opacities	5 (8.2%)
Ground glass infiltration	3 (4.9%)
Rx B (ML)	
Without abnormalities	11 (18.1%)
Interstitial thickening or reticulation	1 (1.6%)
Small opacities	26 (42.6%)
Ground glass infiltration	23 (37.7%)
Rx C (RLL)	
Without abnormalities	26 (42.6%)
Interstitial thickening or reticulation	2 (3.3%)
Small opacities	17 (27.9%)
Ground glass infiltration	16 (26.2%)
Rx D (LUL)	
Without abnormalities	58 (95.2%)
Interstitial thickening or reticulation	1 (1.6%)
Small opacities	1 (1.6%)
Ground glass infiltration	1 (1.6%)
Rx E (L)	
Without abnormalities	11 (18.1%)
Interstitial thickening or reticulation	2 (3.3%)
Small opacities	30 (49.1%)
Ground glass infiltration	18 (29.5%)
Rx F (LLL)	
Without abnormalities	23 (37.7%)
Interstitial thickening or reticulation	2 (3.3%)
Small opacities	23 (37.7%)
Ground glass infiltration	13 (21.3%)
CRX score (median)	7.11 (2–15)
Biomarkers	
D-Dimer (median)	1474 (57–9366)
LDH (median)	385 (130–840)
Lymphocytes (median)	723 (100–1700)

*COPD* Chronic obstructive pulmonary disease. *ICU* Intensive care unit. *IRCU* Intermediate Respiratory Care Unit. *NIMV*: Non-invasive mechanical ventilation. *MV* mechanical ventilation. *CXR score*: Chest X-ray severity score. *RUL* Right upper lobe. *LM* Middle lobe. *RLL* Right lower lobe. *LUL* Left upper lobe. *L* lingula. *LLL* Left lower lobe. *LDH* Lactate dehydrogenase

Details of the EQ-5D questionnaire at 3-months follow-up after discharge are presented in Table 2. Participants showed more problems in the domains of mobility, pain or discomfort, and anxiety/ depression.

**Table 2:** Health-related quality of life of patients with COVID-19 pneumonia 3 months after discharge

	Follow-up questionnaires (3 months) after discharge *n* = 61
Civil state	
Unmarried	4 (6.6%)
Married	55 (90.1%)
Widower/widow	2 (3.3%)
Study level	
Without studies	7 (11.6%)
Primary education	29 (47.5%)
Secondary education	14 (22.9%)
University	11 (18,0%)
EQ 5D	
Mobility	
No problem	41 (67.2%)
Some problems	20 (32.8%)
Extreme problems	0 (0.0%)
Self-care	
No problem	54 (88.5%)
Some problems	7 (11.5%)
Extreme problems	0 (0.0%)
Usual activities	
No problem	57 (93.4%)
Some problems	2 (3.3%)
Extreme problems	2 (3.3%)
Pain/discomfort	
No problem	43 (70.5%)
Some problems	15 (24.6%)
Extreme problems	3 (4.9%)
Anxiety/depression	
No problem	36 (59.0%)
Some problems	17 (27.9%)
Extreme problems	8 (13.1%)
E-VAS (median)	81.5 (25–100)
EQ 5D index (median)	0.77 (0.22–1.00)
Severity index (median)	7.88 (0–30)

*EQ 5D*: EuroQol five-dimension five-level. *E-VAS*: EuroQol Visual Analogue Scale

Clinical, radiological and functional information about the enrolled individuals can be found in Table 3.

**Table 3:** Clinical, radiological and functional follow-up

	First follow-up after discharge (3 months)	Second follow-up after discharge (10 months)	*p*-value
Dyspnoea (mMRC scale)			2.6E-5 (paired-sample *t*-test)
0	30 (49.2%)	48 (78.7%)	
1	9 (14.7%)	8 (13.1%)	
2	17 (27.9%)	4 (6.6%)	
3	5 (8.2%)	1 (1.6%)	
4	0 (0.0%)	0 (0.0%)	
Rx A (RUL)			0.16 (paired-sample *t*-test)
Without abnormalities	58 (95.1%)	60 (98.4)	
Interstitial reticulation	3 (4.9%)	1 (1.6%)	
Small opacities	0 (0.0%)	0 (0.0%)	
Ground glass infiltration	0 (0.0%)	0 (0.0%)	
Rx B (ML)			1.2E-4 (paired-sample *t*-test)
Without abnormalities	40 (65.6%)	52 (85.3%)	
Interstitial reticulation	13 (21.3%)	8 (13.1%)	
Small opacities	8 (13.1%)	1 (1.6%)	
Ground glass infiltration	0 (0.0%)	0 (0.0%)	
Rx C (RLL)			0.011 (paired-sample *t*-test)
Without abnormalities	48 (78.7%)	51 (83.7%)	
Interstitial reticulation	7 (11.5%)	9 (14.7%)	
Small opacities	5 (8.2%)	1 (1.6%)	
Ground glass infiltration	1 (1.6%)	0 (0.0%)	
Rx D (LUL)			0.16 (paired-sample *t*-test)
Without abnormalities	59 (96.7%)	61 (100.0%)	
Interstitial reticulation	2 (3.3%)	0 (0.0%)	
Small opacities	0 (0.0%)	0 (0.0%)	
Ground glass infiltration	0 (0.0%)	0 (0.0%)	
Rx E (L)			0.011 (paired-sample *t*-test)
Without abnormalities	47 (77.0%)	53 (86.9%)	
Interstitial reticulation	10 (16.4%)	7 (11.5%)	
Small opacities	4 (6.6%)	1 (1.6%)	
Ground glass infiltration	0 (0.0%)	0 (0.0%)	
Rx F (LLL)			0.034 (paired-sample *t*-test)
Without abnormalities	46 (75.4%)	50 (82.0%)	
Interstitial reticulation	12 (19.7%)	11 (18.0%)	
Small opacities	3 (4.9%)	0 (0.0%)	
Ground glass infiltration	0 (0.0%)	0 (0.0%)	
CXR score	3.65 (1–8)	1.83 (0–6)	3.8E-6 (paired-sample *t*-test)
Radiological sequelae	25 (41.0%)	17 (27.9%)	5.7E-9 (χ^2^ test)
Pulmonary function tests			
FVC (ml)	3072 (1830–5100)	3266 (1930–5470)	0.039 (paired-sample *t*-test)
FVC (%)	99 (64–145)	109 (69–149)	0.018 (paired-sample *t*-test)
FVC < 80%	6 (9.8%)	2 (3.3%)	1.3E-5 (χ^2^ test)
FEV1 (ml)	2248 (1090–4030)	2334 (1290–4040)	0.16 (paired-sample *t*-test)
FEV1 (%)	94 (54–134)	99 (65–130)	0.088 (paired-sample *t*-test)
DLCO (%)	61 (29–96)	70 (36–101)	8.4E-4 (paired-sample *t*-test)
DLCO < 70%	26 (42.6%)	9 (14.7%)	2.4E-3 (χ^2^ test)

*RUL* Right upper lobe. *LM* Middle lobe. *RLL* Right lower lobe. *LUL*: Left upper lobe. *L* Lingula. *LLL* Left lower lobe. *DLCO* Diffusing capacity of the lungs for carbon monoxide. *FVC* Forced vital capacity. *FEV*1 Forced expiratory volume in the first second

### Assessment at 3 months after discharge

Four different types of response variables were investigated in the initial patient assessment conducted 3 months after discharge: life quality indices, symptom persistence, radiological outcomes and pulmonary function levels. Based on the individual PLS models built for each one of such response variables, specific relationships linking them to the aforementioned regressors (encompassing demographic characteristics, clinical parameters and laboratory test results) were found. The most relevant of these relationships (identified as statistically significant—p-values < 0.05—by both the univariate and the multivariate approaches utilized in this study) are outlined in the following subsections.

#### Life quality indices

**Fig. 1:**
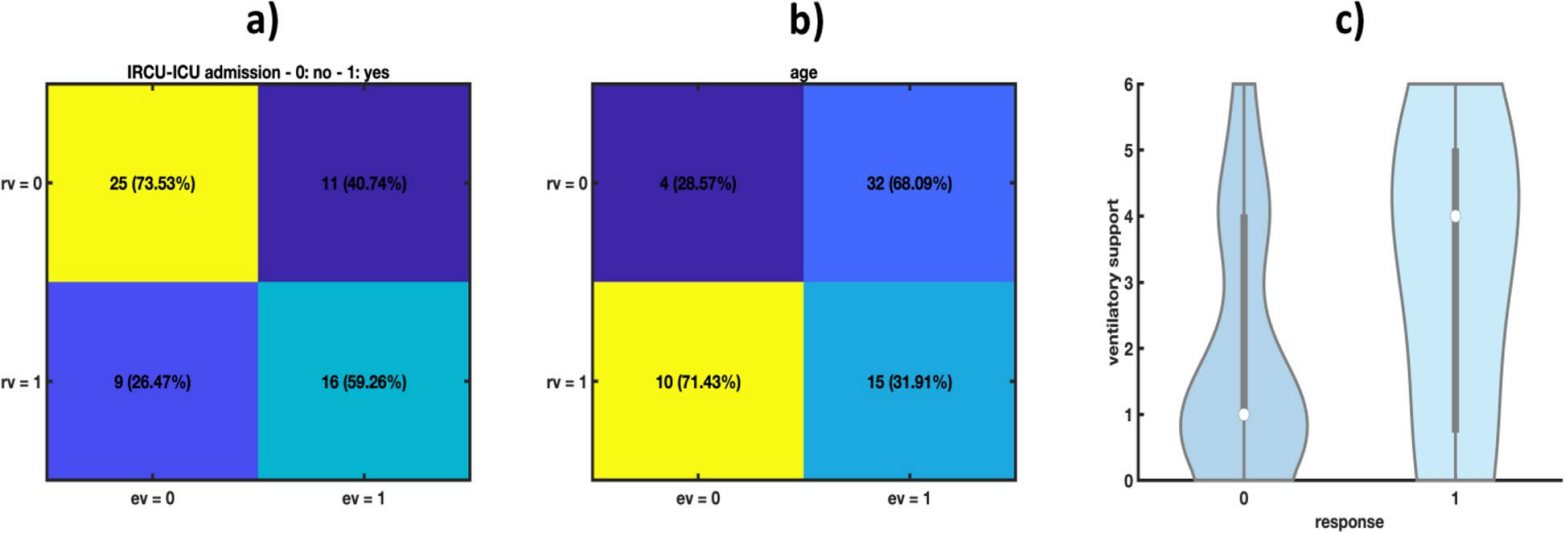
Anxiety/depression symptoms after 3 months from discharge (response variable, *rv*): contingency tables for a) IRCU-ICU admission and b) age (explanatory variables, *ev*. Age was here dichotomized with an optimal threshold set at 57 years; c) violin plot of anxiety/depression symptoms *vs* ventilatory support. The higher the *y*-axis values in c), the more invasive the ventilatory support resorted to

The effect of several predictors on the presence of depression and anxiety symptoms at 3 months after discharge emerged as statistically significant (p-values < 0.05) in the PLS model. More specifically, the obtained results suggest that being a young patient, having been admitted to ICU or IRCU and having needed high-flow oxygen therapy, continuous positive airway pressure, non-invasive mechanical ventilation or mechanical invasive ventilation may increase the risk of occurrence of such symptoms. These conclusions were also corroborated by the univariate assessments conducted for a better understanding of the interrelations between these regressors and the presence of depression symptoms after 3 months from discharge: *p*-values lower than 0.05 were returned for all the χ^2^-tests performed (the predictors as well as the response under study are, in fact, codified as categorical variables). Notice that here also age was dichotomized: the optimal threshold value (57 years) was determined by Kernel Density Estimation (KDE). 40,3% of our population has presented anxiety and depression after 3 months (Fig. 1).

Concerning the occurrence of pain after 3 months from discharge, high blood concentrations of D-dimer (DD) and lactate dehydrogenase (LDH) – both variables were dichotomized as detailed before setting a threshold at approximately 2450 ng/mL and 418 U/L, respectively – were found to constitute risk factors. Univariate χ^2^-tests yielded a *p*-value lower than 0.05 for the two regressors. Around 29% of our cohort has presented pain after 3 months.

**Fig. 2:**
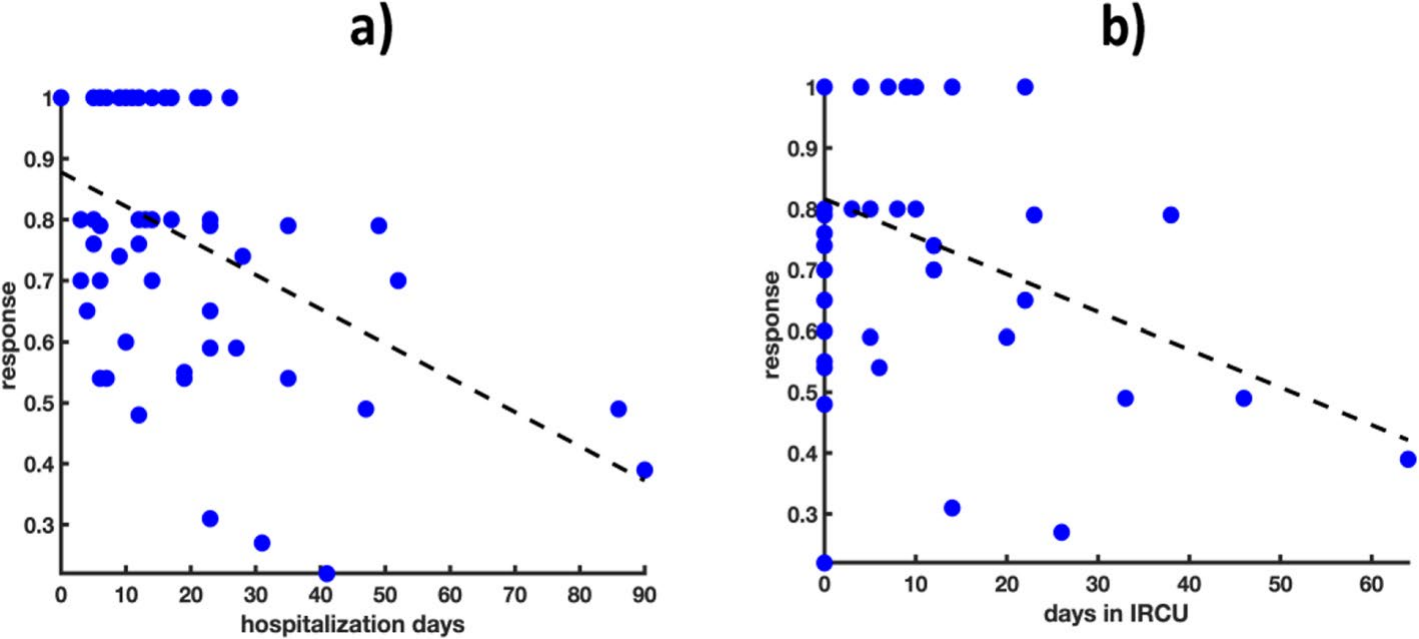
Scatter plots of a) hospitalization days and b) days in IRCU *vs* EQ-5D (response variable). The dashed black lines denote the estimated linear regression functions

**Fig. 3:**
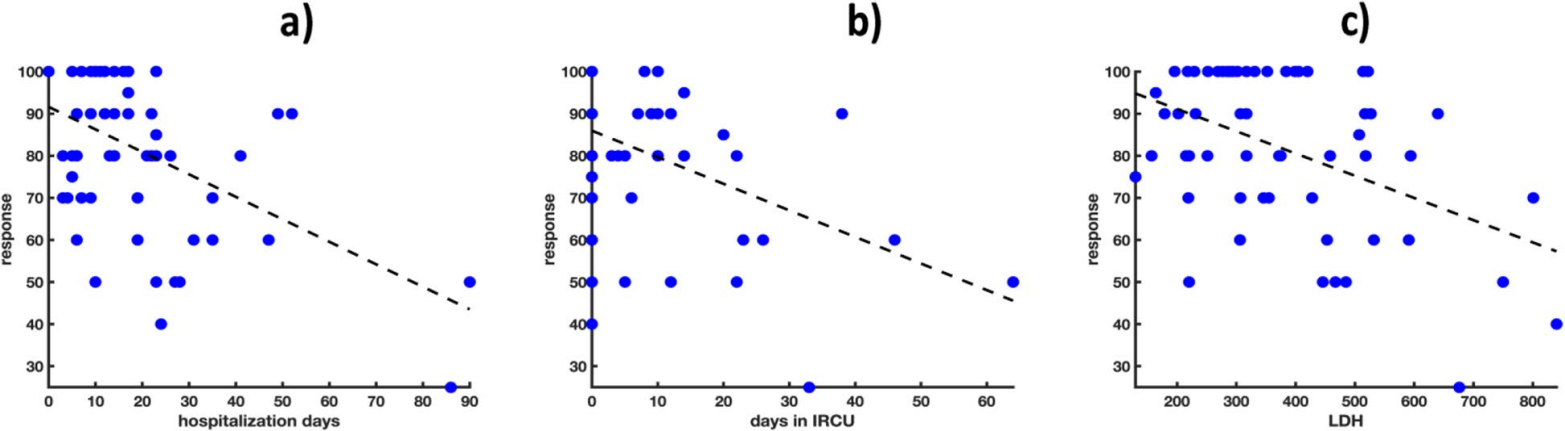
Scatter plots of a) hospitalization days, b) days in IRCU and c) LDH content *vs* VAS (response variable). The dashed black lines denote the estimated linear regression functions

Both the EQ-5D and the VAS global responses were pointed out as negatively correlated to the duration of the patient hospitalization (regression coefficient-associated *p*-value < 0.05), the number of days spent in IRCU units (regression coefficient-associated *p*-value < 0.05). Furthermore, high blood concentrations of LDH (regression coefficient-associated *p*-value < 0.05) was associated with lower VAS response values (Fig. 2 and 3).

**Fig. 4:**
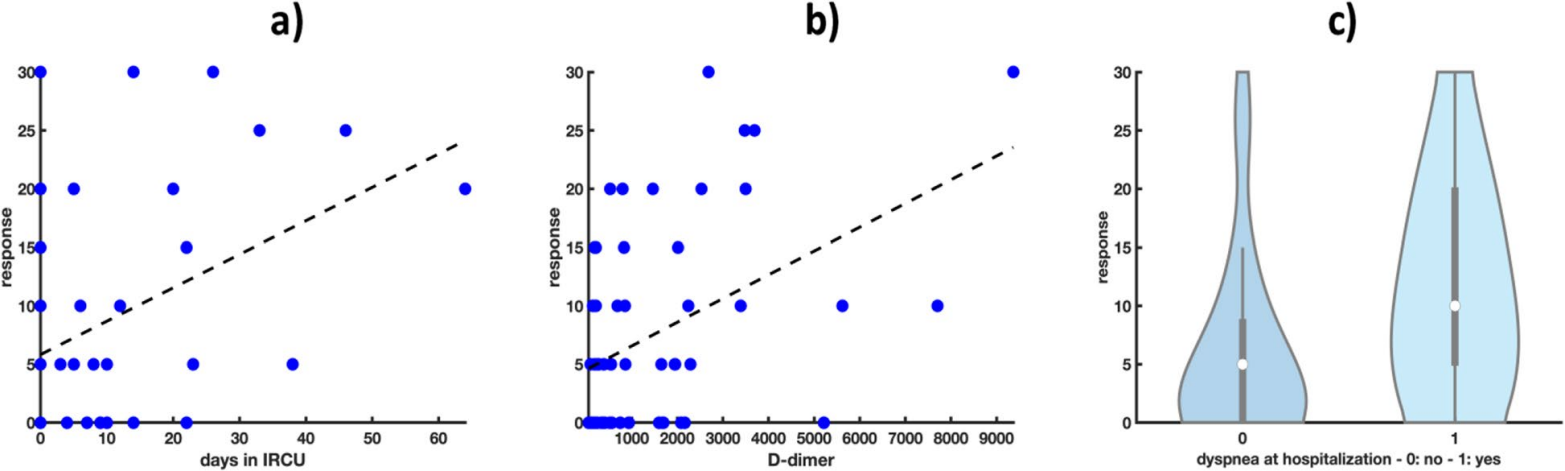
Scatter plots of a) days in IRCU and b) D-dimer content *vs* severity index (response variable). The dashed black lines denote the estimated linear regression functions; c) violin plot of dyspnea at hospitalization *vs* severity index

Finally, the duration of stay in IRCU, dyspnoea at hospitalization and D-dimer content appeared to significantly increase the severity index (regression coefficient-associated *p*-value < 0.05) (Fig. 4).

#### Symptom persistence

The main conclusion one could draw according to the results obtained for this type of response variables regards the fact that patients with evident dyspnoea symptoms at the beginning of their hospitalization show more dyspnoea (mMRC) both after 3 months and 10 months from discharge in comparison to those which had less dyspnoea at admission. Moreover, dyspnoea (mMRC) at the long-term follow-up is associated with a lower EQ-5D score.

#### Radiological outcomes

The overall radiological outcome at 3 months after discharge was found to be statistically significantly influenced by the severity of pulmonary affectation at the beginning of the hospitalization period (more specifically to the level of pulmonary affection at the middle and inferior lung lobes – regression coefficient-associated *p*-values < 0.05).

#### Pulmonary function levels

Lower FVC at 3 months is significantly associated with male sex (ANOVA *p*-value < 0.05), older age, lymphocyte content and longer IRCU stay (regression coefficient-associated *p*-values < 0.05). Conversely, DLCO (dichotomized based a threshold set at 70%) was found to be negatively correlated to lymphopenia (lymphocytes content was here dichotomized according to a threshold set at approximately 816 × 10^6^/L – χ^2^-test *p*-value < 0.05).

### Assessment at 10 months after discharge

#### Symptom persistence

After 10 months from discharge, dyspnoea symptoms tend to persist in patients that exhibited lower EQ-5D and VAS life quality indices at 3 months after discharge (regression coefficient-associated *p*-values < 0.05).

#### Radiological outcomes

Long-term pulmonary affectation was mostly observed in men (χ^2^-test *p*-value < 0.05) and for individuals whose radiological assessment carried out 3 months after discharge had already highlighted specific issues. More radiological abnormalities were detected in lower lobes than in upper lobes (see Table3) and the score was lower in lung upper lobes (*t*-test *p*-value < 0.05).

#### Pulmonary function levels

Low FVC values were detected 10 months after discharge for subjects exhibiting high levels of dyspnoea (regression coefficient-associated *p*-value < 0.05), and low FVC (regression coefficient-associated *p*-value < 0.05) at 3 months after discharge. Moreover, FVC at long term (dichotomized based a threshold set at 80%) was related with high blood concentrations of ferritin (dichotomized according to a threshold set at approximately 4016 ng/mL – χ^2^-test *p*-value < 0.05) and lymphopenia (lymphocytes content was here dichotomized according to a threshold set at approximately 317 × 10^6^/L – χ^2^-test *p*-value < 0.05).

**Fig. 5:**
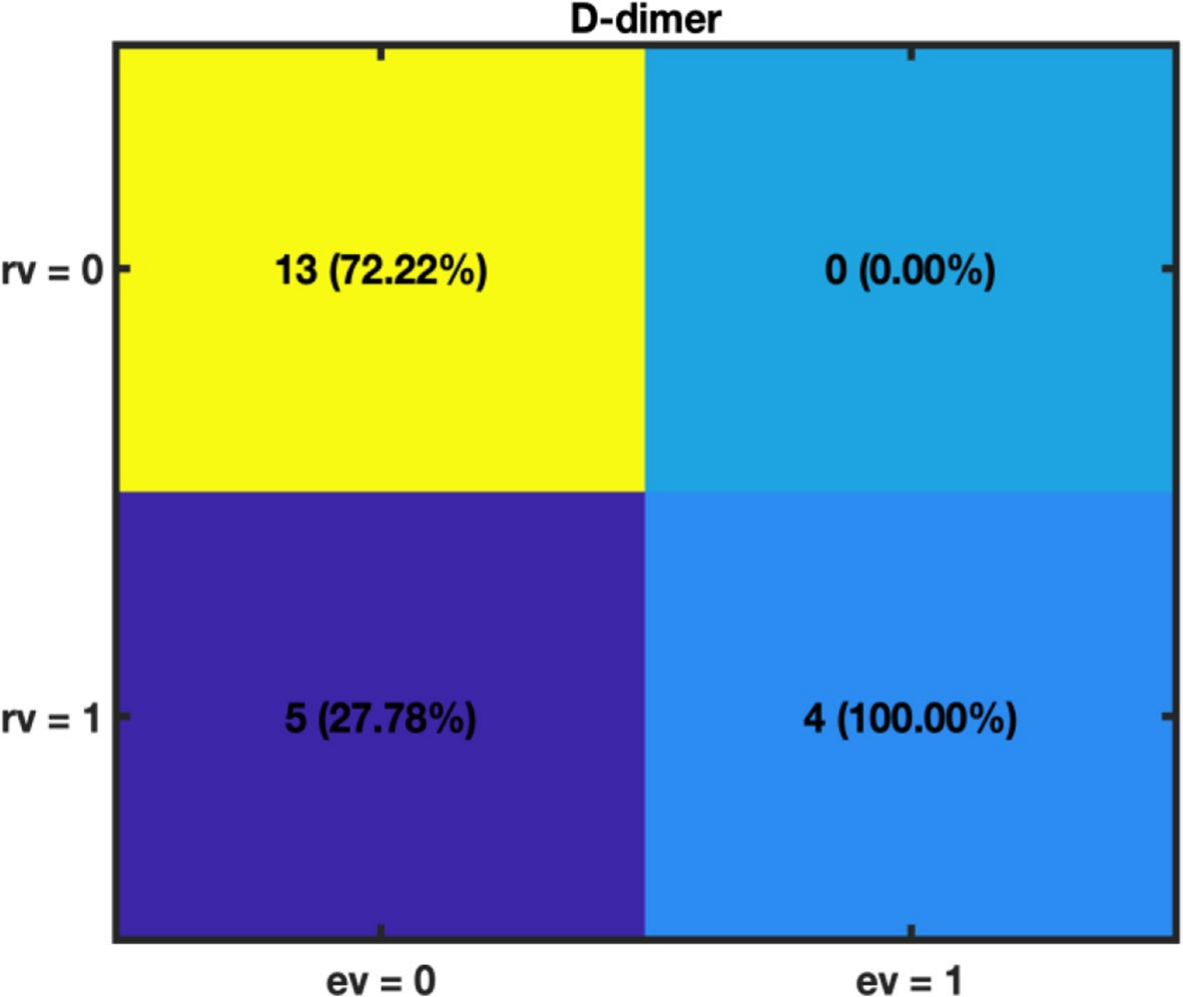
DLCO < 70% after 10 months from discharge (response variable, *rv*): contingency table for D-dimer (explanatory variable, *ev*). D-dimer content was here dichotomized with an optimal threshold set at 3212 ng/mL

DLCO < 70% at 10 months after discharge was pointed out as positively correlated to DLCO < 70% at 3 months after discharge and higher levels of D-dimer (dichotomized according to a threshold set at approximately 3212 ng/mL—χ^2^-test *p*-value < 0.05) (Fig. 5).

## Discussion

Since SARS-CoV-2 outbreak in March 2020, several studies have been carried out to analyse physical, mental and pulmonary consequences in patients with COVID-19 infection after discharge.

The present study aimed to assess the quality of life in our population after an average observation time of 3 months. Our cohort reveals that the mean EQ-5D and E-VAS index scores decreased with hospitalization time, the number of days spent in IRCU units and the level of dyspnoea at the beginning of the hospitalization period. A lower average in E-VAS is also associated to higher LDH serum levels.

The previous outbreaks of SARS and MERS used SF‐36 to measure QoL, and showed a significantly low quality of life at 1 year in affected patients, lower than the quality of life of those with chronic pathologies (using normative data) [2].

Current literature suggests similar postdischarge symptoms. Halpin et al. found that patients admitted to ICU had a greater prevalence of symptoms in almost all reported symptom domains, despite being a younger and less comorbid group [17]. This is in keeping with the well‐ characterized post‐intensive care syndrome [18].

Anxiety/depression was the most frequently reported symptom (40,3% of our population). Those who are younger than 57 dropped to a level of below average, and it is correlated with ICU/IRCU admission, the need of ventilatory support and the need of oxygen therapy after discharge.

Two Chinese studies found that anxiety or depression were common, even at 6 months follow-up after symptom onset, being the severity of illness a risk factor for persistent psychological symptoms [19, 20]. The underlying mechanism of the psychiatric consequences of COVID-19 is likely to be multifactorial and might include the direct effects of viral infection, the immunological response, corticosteroid therapy, ICU stay and social isolation [21].

Mobility and pain were other reported problems in a proportion close to anxiety/depression. We found that pain was associated with higher levels of LDH and D-dimer, which is consistent with other studies which found a correlation between pain/discomfort and clinical severity [22, 23].

Breathlessness was a significant problem both after 3 month and 10 months after discharge. Those which presented more dyspnoea at admission have greater dyspnoea at 3 months (measured by mMRC scale). After 10 months, those who had more dyspnoea at admission continued to have greater dyspnoea, as well as those who had a poorer quality of life (poorer result in EQ5D index). Regarding dyspnoea on exertion (by Borg) after 10 months, it is associated with those who spent more days in ICU, higher severity index and worse quality of life measured 3 months after hospital admission.

Our results are comparable to those demonstrated in a multicentre cross-sectional cohort study in Spain, which reports that dyspnoea as persistent symptom was significantly associated with number of days at hospital, number of medical comorbidities and number of symptoms at hospitalization [24]. Another study described that those needing ICU admission and respiratory support, premorbid lung problems, higher age, higher BMI, and BAME ethnicity are more likely to experience breathlessness postdischarge [15], being comparable to those reported in the meta‐analysis study of 11% to 45% of survivors having breathlessness even up to one year [2].

In this sense, the implementation of IRCUs is a useful alternative to increase the availability of ICU beds while maintaining high-quality care, contributing to a faster recovery process, since it provides specific physiotherapy and improves patient care by having a higher doctor-patient/nurse-patient ratio than is available in a conventional ward. Thus, a IRCU provides multidisciplinary care that shortens ICU time and potentially the overall length of hospital stay [25].

We also analysed the radiological features after 3 and 10 months. Radiological abnormalities in patients 10 months after discharge were more frequent in those which had previous higher CXR scores (at admission and at 3 months follow-up) and a higher value in mMRC dyspnoea scale. Therefore, a poorer result in CXR score at 3 months precedes long-term pulmonary sequelae.

In the follow-up of severe COVID-19, imaging techniques play a fundamental role. It became necessary to develop radiological tools that can predict which patients are at higher risk of developing rapid and fatal disease progression during the acute phase. Severity scores based on chest radiography have been proposed to stratify patients by quantifying the extent of disease and to predict both the need for ICU referral and death on hospital admission [26].

An Italian study including 119 patients showed that the radiological abnormalities were present in about half patients 3 months after discharge, which had higher age, previous higher CXR scores and longer hospitalization. Days of hospitalization and previous CXR scores were independent factors for predicting the CXR at three months [27]. Based on a scoring system the authors used, they suggested this finding could help prioritize patients with more severe clinical and radiological findings.

Recent studies suggest different techniques that could provide a powerful monitoring aid in patients with suspected COVID-19 pneumonia, such as chest ultrasound or compliance measurement with forced oscillometry, which could anticipate radiological findings and consequently patients may benefit from an early initiation of non-invasive ventilation [26].

Several factors have been correlated with the risk of developing fibrosis and persistent functional deficits in COVID-19: severity of infection, need for ICU admission and mechanical ventilation, older age, higher body mass index, and smoking history [26] Therefore, patients who recover from SARS-CoV-2-related ARDS but experience persistent respiratory symptoms should undergo radiological follow-up to determine whether there are abnormalities that may anticipate the establishment of fibrosis.

It has been proposed that after 3 months is a good time to perform follow-up and assess the resolution of this condition [28].

The results of lung function assessment in our study showed that a considerable part of the participants had abnormalities regarding lung capacity and diffusion. We found that higher average at mMRC scale at 3 months is associated with a lower FVC in a long-term follow-up (10 months) and predisposing factors for a FVC < 80% after 10 months are the high levels of ferritin and lymphopenia at admission. Regarding DLCO, high D-dimer levels on admission predict poorer long-term diffusion capacity (DLCO < 70%).

Zhao et al. reported that at 3-months after discharge, residual abnormalities of pulmonary function were observed in around 25% of their cohort, mostly demonstrated diffusion reductions in DLCO [7]. In the following-up studies for the patients rehabilitating from SARS, impaired lung function could last for months or even years [29]. At 6, 9 and 12 months after discharge, the same phenomenon can be noted [30].

D-dimer elevation has been determined as an important laboratory finding in COVID-19 patients. The level of D-dimer was an important prognostic factor for abnormal DLCO. Thus, for those patients who have high D-dimer content, pulmonary rehabilitation should be needed subsequently [7], whereas Méndez et al. and Sibila et al. observed that patients with impaired DLCO also showed 3 months after discharged higher values of C-reactive protein (CRP), D-dimer and lactate dehydrogenase (LDH) [31, 32], which is consistent with the data of our study. In addition, prolonged ICU and hospital stay and breathlessness were also associated with reduced DLCO [30].

A recent study states that patients with invasive mechanical ventilation had higher inflammation levels than those with non-invasive ventilation, which may indicate that cytokine patterns could be useful as a prognostic tool in terms of anti-inflammatory therapy (i.e.corticosteroids) tayloring and timely iniciation of invasive mechanical ventilation [33].

In the Swiss national cohort, lower lung volumes (FVC and FEV1) 4 months after severe/critical COVID-19 were demonstrated in several patients; the higher FEV1/FVC ratio in the severe/critical subgroup suggests a tendency toward a restrictive physiology, and there was a negative correlation between the duration of mechanical ventilation during the acute disease and pulmonary function at 4-month follow-up [34].

However, there are several limitations in this study. It involves a limited number of participants who do not constitute a representative example of the population. Furthermore, it does not include COVID‐19 survivors who were not hospitalized and it is likely that non‐hospitalized COVID‐19 survivors will have different rehabilitation needs to those who were hospitalized, so this needs further investigation. Moreover, the presence of different comorbidities in some of our patients may have impacted on symptoms reported in quality of live questionnaires. We carried out a single center study with a limited number of cases, being difficult to assess risk factors for disease severity and mortality with multivariable adjusted methods.

## Conclusions

To conclude, after 3 months follow-up, the clinically significant drop seen in our cohort reflects the impact of the illness on quality of life. COVID-19 related breathlessness and psychological distress were commonly reported with greater prevalence in those needing ICU care. Rehabilitation care for COVID‐19 survivors must, therefore, be consider to meet the needs of these patients. Rehabilitation starts with a diagnostic process aiming to discover what the patient's concerns are and to understand how they arise and may be afforded. In this context, the attachment of a Rehabilitation Unit to a respiratory medicine department may be a priority in COVID-19 patients not only at hospital stay but also after discharge, in order to improve patients disabilities with effective rehabilitation interventions and plannings.

Both chest x-ray and pulmonary function test may be helpful as an aiding tool in the diagnosis and follow up in patients with COVID-19 pneumonia, as they could play a central role in mid to long-term follow-up of COVID-19, assessing the radiological and functional sequelae of patients and identifying those who might require a closer follow-up.

Longer follow-up studies in a larger population are necessary to be carried out to obtained further data about consequences related to COVID-19.

## Data Availability

The datasets generated and/or analysed during the current study are not publicly available due to the non-translation of the dataset into English language, but are available from the corresponding author on reasonable request.

## Abbreviatures

HRQoL: Health-related quality of life
EQ-5D: EuroQol five-dimension
E-VAS: EuroQol Visual Analogue Scale
QoL: Quality of life
ICU: Intensive care unit
IRCU: Intermediate respiratory care unit
FVC: Forced vital capacity
DLCO: Diffusing lung capacity of carbon monoxide
SARS-CoV-2: Severe acute respiratory syndrome coronavirus 2
COVID-19: Coronavirus disease 2019
SARS: Severe acute respiratory syndrome
MERS: Middle East respiratory syndrome
ARDS: Acute respiratory distress syndrome
CXRs: Chest X-ray
ATS: American Thoracic Society
ERS: European Respiratory Society
PLS: Partial Least Squares regression
RMSECV: Root Mean Square Error in Cross-Validation
AUROC: Area Under the Receiver Operating Curve
KDE: Kernel Density Estimation
DD: D-dimer
LDH: Lactate dehydrogenase.
mMRC: Modified MEDICAL RESEARCH COUNCIL
BMI: Body mass index
CRP: C-reactive protein
